# Evaluation of a Digital Diabetes Prevention Program Adapted for Low-Income Patients, 2016–2018

**DOI:** 10.5888/pcd16.190156

**Published:** 2019-11-27

**Authors:** Sue E. Kim, Cynthia M. Castro Sweet, Edward Cho, Jennifer Tsai, Michael R. Cousineau

**Affiliations:** 1 Department of Preventive Medicine, Keck School of Medicine, University of Southern California, Los Angeles, California; 2 Omada Health, Incorporated, San Francisco, California; 3 Gehr Family Center for Health Systems Science and Innovation, Keck School of Medicine, University of Southern California, Los Angeles, California

## Abstract

**Introduction:**

We examined the effects of a digitally delivered, type 2 diabetes mellitus prevention program (DPP) for a low-income population.

**Methods:**

We conducted a nonrandomized clinical trial with matched controls. The intervention group was offered a digital DPP, a web-based and mobile-based program including 52 weeks of participation in an educational curriculum, health coaching, and peer support.

**Results:**

A total of 227 participants enrolled. At baseline, 34.6 was the mean body mass index, and 5.8 was the mean HbA_1c_. For the intervention group, mean weight loss was 4.4% at the 12-month follow-up.

**Conclusion:**

The modified DPP successfully engaged participants and resulted in weight loss. Low-income patients with prediabetes benefitted from a digitally delivered diabetes intervention. This prevention method should be accessible to a low-income population.

**Summary**

**What is already known on this topic?**

Diabetes Prevention Programs (DPPs) demonstrate that lifestyle changes can be more effective than prescription medication to prevent or delay the onset of diabetes. Although DPPs have focused on low-income communities and achieved promising results, in-person participation continues to be a challenge.

**What is added by this report?**

Our study examined a digitally delivered DPP that removed some barriers to access and increased participation for low-income patients.

**What are the implications for public health practice?**

We revealed that a digitally delivered intervention, allowing choice of access, can be an effective option in preventing or delaying diabetes by increasing participant engagement.

## Introduction

In 2015, 84 million US adults were estimated to have prediabetes and approximately 30 million were living with diabetes (1). While diabetes prevention efforts increase, the incidence of type 2 diabetes mellitus and obesity remain disproportionately higher among low-income patients, including those from underrepresented racial and ethnic groups (2,3).

The landmark Diabetes Prevention Program (DPP) demonstrated that lifestyle modifications improve healthy diet, increase physical activity, and sustain weight loss more effectively than prescription medication in preventing or delaying the onset of diabetes (4,5). The success of the DPP lifestyle intervention highlights the role of behavioral interventions as effective, safe, and sustainable for diabetes prevention (6–8). Translational efforts have disseminated the DPP through in-person groups, as well as online and digital formats through remote coach access, internet platforms, telecommunications, and smartphone apps, resulting in replication of DPP goals (8,9).

Some DPPs have targeted low-income communities with modest but promising results (10–14). Recurring limitations in many of these programs are dependency on face-to-face interactions, location-based meetings, and time-restricted options for group sessions. Limited flexibility of work schedules, access to reliable transportation, and access to affordable childcare are reported obstacles for participation in required, in-person DPP sessions (15,16). Similarly, people in rural areas might live long distances from the nearest DPP location, posing a transportation challenge.

Given low-income communities’ growing use and acceptance of accessible technologies (17,18), a digitally delivered DPP might be an option for hard-to-reach populations with prediabetes (19). We examined the effectiveness of a digital DPP adapted for a low-income population. We designed the study to measure participation in the program and its effectiveness in reducing risk for diabetes, compared with a nonparticipating matched group.

## Methods

We conducted the intervention at a federally qualified health center and outpatient clinic in a large public teaching hospital in Southern California and at a clinic within a private, integrated health care network in Washington State from February 2016 through April 2018. The target population was adults (aged 18–75 years) enrolled in Medicaid or another safety-net insurance plan, and with evidence of prediabetes in their electronic health record (EHR). Our previous publication describes implementation of the digital DPP for a low-income population, including study design, protocol, and inclusion and exclusion criteria (20). We registered this study at ClinicalTrials.gov (NCT02664064), and our analyses are based on the previous protocol. The DPP used in this study achieved Full Recognition status from the Centers for Disease Control and Prevention’s Diabetes Prevention Recognition Program (21). Both the Western Institutional Review Board and the Health Sciences Institutional Review Board at the University of Southern California reviewed and approved this study’s protocol.

We identified participants through EHRs or by referrals from their primary care physicians. In total, we identified and screened 273 participants who were eligible for inclusion in the study, and a digital DPP was offered to all in the intervention group (Figure).

**Figure:**
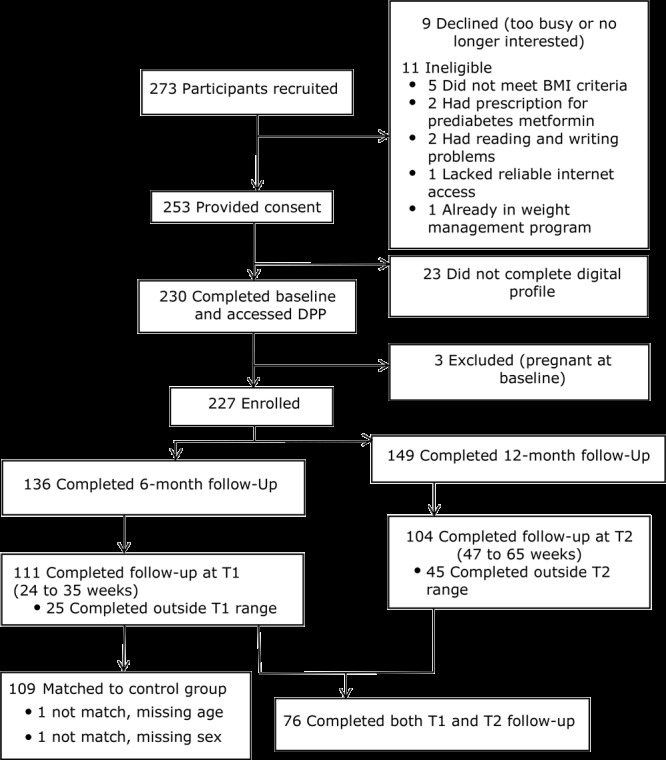
Participant selection for digitally delivered Diabetes Prevention Program (DPP). Abbreviations: BMI, body mass index; DPP, Diabetes Prevention Program.

The intervention is a digital DPP that includes virtual group support, personalized health coaching, weekly lessons, and digital progress tracking tools (20,22). The program begins with an intensive 16-week phase, followed by a 36-week maintenance phase. Participants are assigned to small virtual groups with peers and a health coach. Each group has a private online social network to engage in discussion and provide social support (20). Participants complete weekly health education lessons available on the digital platform that can be accessed through the internet or smartphones. Health coaches communicate with participants to provide individual counseling through private messaging and facilitate group discussions through a chat board. The DPP curriculum was adapted for low-income populations by rewriting content at 4th- and 5th-grade reading levels, cultural tailoring and Spanish translation, and adding bilingual and bicultural health coaches. These adaptations were previously tested in a feasibility study (20,23).

### Outcome measures

At baseline, study site coordinators recorded demographic data (marital status, household size, sex, race/ethnicity, preferred language, educational status, and current employment status), insurance status, self-rated health, self-efficacy for diet and physical activities, health beliefs, health care utilization, self-management, comfort with technology, health literacy, and social support. We collected these measures only for participants who received the intervention; matched controls did not provide this information. Because not all participants were available for the 6-month or 12-month follow-up at the same time, and to avoid overlapping times between the 2 follow-up periods, we defined the first follow-up time as T1 to approximate 6 months after baseline and included data collected from 24 to 35 weeks. Time T2 approximates 12 months after baseline and included data collected from 47 to 65 weeks. We determined these points on sensitivity analyses of the distribution and natural break when the majority of participants completed each of the follow-ups. With the exception of demographics, we repeated all measures in both interviewer-administered surveys at T1 and T2 (20).

Using calibrated equipment in the health clinics, we measured height using stadiometers and weight using weight scales at baseline, T1, and T2. The primary outcome variable was change in weight over time, both in pounds and as percentage change. In assessing change in the comparison group, we used change in body mass index (BMI, calculated as weight in kilograms divided by height in meters squared [kg/m^2^]), because one of the study sites did not provide separate weight and height data. Using self-administered AccuBase (DTI Laboratories) A_1c_ finger-stick test kits, we recorded glycosylated hemoglobin (HbA_1c_) in percentage units of NGSP (formerly, National Glycohemoglobin Standardization Program) and DCCT (Diabetes Control and Complications Trial). We measured and defined engagement as the number of completed lessons in the initial 16-week phase, with a range of 0 to 16.

### Matched control group

Each study site provided control group data from de-identified records of patients who did not enroll in the trial, and each site used the same criteria for study eligibility (24). We matched the control group on age and sex. Each site searched for matched control cases within 12 months before the start of enrollment or concurrent with the enrollment period of the trial. This allowed a maximum 24-month window to find a matched control group with at least 2 measurements of BMI and HbA_1c_ test results.

To create a control group that best matched the intervention group, we used a one-to-many matching algorithm. We first identified all possible matches for each intervention participant by setting exact match criteria for sex and site location. To have at least 1 control group match for each intervention case, we set participant age and intervals between BMI measurements flexibly, with a range of 15 years and 45 days, respectively. We determined the flexible matching criteria to optimize having at least 1 control match to an intervention participant (24).

### Statistical analyses

We set the study to detect a significant predifference and postdifference of 3% weight loss in the intervention group relative to a 0% weight reduction expected in the control group. Based on a similar study with comparable populations (25), we estimated a 4% standard deviation (SD) across groups. With α = .05 and power = 0.8, we estimated a minimal sample size of 40 participants per group necessary to detect a 3% difference in weight loss. Estimated churn rates for Medicaid varied between 20% and 50% (26,27), from which we expected a 30% and 40% baseline loss to follow-up rate.

We examined weight loss as the primary outcome, as measured by mean percentage weight loss from baseline to T1 and T2, as well as the percentage who lost more than 5% of their baseline weight and change in BMI from baseline to T1 and T2. As a process measure, we examined level of participation in the DPP, as measured by program engagement (defined as lesson completion) and changes in HbA_1c_ measurement from baseline to T1 and T2. Because of the number of participants who did not complete the study and were excluded, we conducted an attrition analysis to determine if biases were present in the sample of those who did complete T1 and T2 assessments. We compared the demographic characteristics of participants among 5 subgroups: the overall sample of participants at baseline, participants with T1 follow-up data, participants with T2 follow-up data, participants without T1 data, and participants without T2 data.

We measured weight change at times T1 and T2 as a continuous variable (percentage weight change). For the bivariate analyses, we dichotomized the outcomes to 5% or less and above 5%. To compare our findings to previous evaluations of DPP, we chose the benchmark of 5% and examined outcomes anchored on lesson completion (9). To test associations between key demographics and predictor variables for percentage change in weight, we created 3 regression models: 1) a continuous variable, 2) the odds of losing more than 5% of baseline weight, and 3) engagement. Covariates were age, sex, race/ethnicity, insurance status, education, employment, self-rated health, and self-efficacy at baseline. For multivariate analyses, we examined ordinary least square regression models. Data analyses were performed using Stata 15 (StataCorp, LLC).

## Results

Study recruitment, screening, and enrollment occurred during February 2016 through March 2017. Participants at baseline (n = 227) were predominantly women (81.3%), average age was of 48.2 years, and 117 (51.5%) identified their ethnicity as Hispanic/Latino (Table 1). A total of 106 (47.1%) indicated Spanish as their preferred language, and 97 (43%) reported limited English proficiency.

**Table 1:** Participant Demographics in Digital Diabetes Program at Baseline and Each Follow-Up^a^

Demographics **c**	Baseline	T1 Participants^b^		T2 Participants^b^		Full Cohort
	All Participants (n = 227)	With Follow-Up Data (n = 111)	Without Follow-Up Data (n = 116)	With Follow-Up Data (n = 104)	Without Follow-Up Data (n = 123)	Baseline, T1, and T2 (n = 76)
**Age, mean (standard deviation), y**	48.2 (11.7)	49.5 (10.6)	47.0 (12.6)	48.8 (10.6)	47.6 (12.5)	50.2 (9.9)
**Female**	183 (81.3)^c^	90 (81.8)	93 (80.9)	81 (78.6)	102 (83.6)	61 (81.3)
**Race/ethnicity**						
Non-Hispanic white	37 (16.3)	22 (19.8)	15 (12.9)	20 (19.2)	13.8 (17)	17 (22.4)
Hispanic/Latino	117 (51.5)	58 (52.3)	59 (50.9)	54 (51.9)	63 (51.2)	38 (50.0)
African American	16 (7.1)	11 (9.9)	5 (4.3)	7 (6.7)	9 (7.3)	6 (7.9)
Other or preferred not to answer	56 (25.0)	20 (18.0)	37 (31.9)	23 (22.1)	34 (27.6)	15 (19.7)
**Language preference**						
English	119 (52.9)	56 (50.9)	63 (54.8)	54 (52.9)	65 (52.9)	39 (52.0
Spanish	106 (47.1)	54 (48.6)	52 (45.2)	48 (47.1)	58 (47.2)	36 (48.0)
**Insurance** c ^,^ d						
Medicaid or Medi-Cal	84 (37.0)	49 (44.1)	35 (30.2)	49 (47.1)	35 (28.5)	39 (51.3)
Other program for uninsured	143 (63.0)	62 (55.9)	81 (69.8)	55 (52.8)	88 (71.5)	37 (48.7)
**Education**						
Some high school	86 (38.4)	42 (37.8)	44 (37.9)	40 (38.8)	46 (37.4)	31 (40.8)
High school diploma or GED	46 (20.5)	25 (22.5)	21 (18.1)	24 (23.3)	22 (18.0)	17) (22.4)
Some college or college graduate	92 (41.1)	44 (39.6)	48 (41.3)	39 (37.9)	53 (43.1)	28 (36.8)
**Employment** e						
Unemployed	104 (47.1)	59 (55.1)	45 (39.5)	51 (50.0)	53 (44.5)	41 (55.4)
Employed part-time^f^	51 (23.1)	25 (23.4)	26 (22.8)	25 (24.5)	26 (21.9)	17 (23.0)
Employed full-time^f^	66 (29.9)	23 (21.5)	43 (37.7)	26 (25.5)	40 (33.6)	16 (21.1)
**Self-rated health at baseline**						
Excellent or very good	117 (55.9)	55(56.7)	62 (55.4)	54 (56.0)	63 (53.9)	36 (55.4)
Good	71 (34.0)	36 (37.1)	35 (31.3)	30 (32.6)	41 (35.0)	24 (36.9)
Fair or Poor	21 (10.1)	6 (6.2)	15 (13.4)	8 (8.7)	13 (11.1)	5 (7.7)
**Self-efficacy composite score at baseline** g, **mean (SD)**	41.8 (7.8)	41.1 (8.3)	42.4 (7.4)	42.2 (8.1)	41.5 (7.7)	42.0 (8.3)
**BMI (weight in kg/height in square meters) at baseline**						
Normal (18.5–<25)	9 (4.0)	3(2.7)	6 (5.2)	5 (4.8)	4 (3.3)	3 (4.0)
Overweight (25–<30)	66 (29.1)	35 (31.5)	31 (26.7)	26 (25.0)	40 (32.5)	22 (29.0)
Obese (≥30)	152 (67.0)	73 (65.8)	79 (68.1)	73 (70.2)	79 (64.2)	51 (67.1)

Abbreviations: BMI, body mass index; GED, general education diploma; SD, standard deviation.

a All numbers are indicated as number and percentage, unless otherwise stated.

b T1, data collected at approximately 6 months after baseline or 24 to 35 weeks; T2, data collected at approximately 12 months after baseline or 47 to 65 weeks.

c Percentages may not add to 100% because of rounding and missing data.

d Insurance: T1, *P* = .03; T2, *P* = .004; and full cohort, *P* = .002, determined by χ^2^ test.

e Employment: T1, *P* = .02, determined by χ^2^ test.

f Employment full-time, >40 hours per week; employment part-time <40 hours per week.

g Self-efficacy composite score for diet and physical activity; sum of 13 items scored from 13 to 52 (each item scored as 1, confident; 2, little confident; 3, somewhat confident; and 4, completely confident).

At baseline, 69 (30.4%) participants had a normal HbA_1c_ (mean 5.3%; SD, 0.2%) and 7 (3.1%) participants had an HbA_1c_ in the diabetes range (mean, 6.6%; SD, 0.1%). Mean BMI was 34.6 (SD, 7.9), mean weight was 199.5 lb (SD, 55.6), and median weight was 190 lb. Although all participant EHR laboratory reports indicated prediabetes, defined as HbA_1c_ level of 5.7%–6.4% in the preceding 6 months, baseline mean HbA_1c_ test results indicated 5.8% (SD, 0.4%).

In our comparison of the 5 subgroups, including groups of participants lost to follow-up, we found that the groups were similar in nearly all sociodemographic variables except insurance status and employment. Those lost to follow-up were less likely to have Medicaid at T1 (*P* = .03) and T2 (*P* = .004) and more likely to work full-time (*P* = .02) at T1. No other significant differences were observed, indicating that those who were lost to follow-up were sociodemographically similar to those who completed follow-up.

In the weight-loss analyses for the 111 participants with complete data at T1 and 104 participants with complete data at T2, we found that overall, 45 (41%) of the participants had more than 5% weight loss at T1 and 38 (37%) had more than 5% weight loss at T2. Mean weight loss was 4.2% (SD, 6.6%) at T1 and 4.4% (SD, 7.7%) at T2 (*P* < .001 for each). Mean BMI at T1 was 33.3, a change of −1.5 from baseline (SD, 2.4; *P* < .001) and the mean at T2 was 33.2, a change of −1.6 from baseline (SD, 2.7; *P* < .001). We used BMI for the comparison analysis because separate weight change data were not available for the control group.

In the bivariate analysis, we compared the results of participants who achieved less weight loss (≤5%) with participants who achieved more weight loss (>5%) from baseline to T1 and to T2. At both T1 and T2, completing 9 or more lessons was significantly associated with weight loss (T1, *P* = .001; T2, *P* = .003) (Table 2). No difference in weight loss was associated with any sociodemographic factors, self-reported health at baseline, BMI at baseline, or HbA_1c_ level at baseline.

**Table 2:** Participant Weight Loss by Demographic Characteristic at Each Follow-Up

Characteristic	T1			T2		
	Pounds Lost (n = 111)^a^	≤5% Body Weight Lost (n = 66)^b^^,^ c	>5% Body Weight Lost (n = 45)^b^^,^ c	Pounds Lost (n = 104)^a^	≤5% Body Weight Lost (n = 66)^b^^,^ c	>5% Weight Lost (n = 38)^b^^,^ c
**Age, no. of particiants (mean in years)[SD]**	NA	65 (48.4) [11.3]	45 (51.0) [9.3]	NA	66 (48.8) [10.2]	37(48.7) [11.5]
**Sex**						
Male	20 (14) [16.8]	9 (45.0)	11 (55.0)	22 (10.2) [13.1]	15 (68.2)	7 (31.8)
Female	90 (7.2) [13.2]	56 (62.2)	34 (37.8)	81 (8.2) [15.5]	50 (61.7)	31 (38.3)
**Race/ethnicity**						
Non-Hispanic white	22 (12.1) [16.1]	10 (45.5)	12 (54.6)	20 (16.6) [16.2]	8 (40.0)	12 (60.0)
Hispanic/Latino	58 (7.5) [14.1]	37 (63.8)	21 (36.2)	54 (5.6) [12.1]	38 (70.4)	16 (29.6)
African American	11 (5.6) [7.6]	6 (54.6)	5 (45.5)	7 (2.4) [16]	5 (71.4)	2 (28.6)
Other/preferred not to answer	20 (8.4) [14.4]	13 (65.0)	7 (35.0)	23 (10.1) [17.2]	15 (65.2)	8 (34.8)
**Language preference**						
English	56 (9.7) [16.4]	32 (57.1)	24 (42.9)	54 (10.7) [17.0]	31 (57.4)	23 (42.6)
Spanish	54 (7.2) [11.3]	33 (61.1)	21(38.9)	48 (6.1) [12.3]	34 (70.8)	14 (29.2)
**Insurance**						
Medicaid or Medi-Cal	49 (15.4) [−7.3]	32 (65.3)	17 (34.7)	49 (15.9) [−7.5]	34 (69.4)	15 (30.6)
Other program for uninsured	62 (13.0) [−9.3]	34 (54.8)	28 (45.2)	55 (14.2) [−9.4]	32 (58.2)	23 (41.8)
**Education^d^**						
Some high school	42 (12.4) [−7.4]	27 (64.3)	15 (35.7)	40 (12.6) [−9.7]	24 (60.0)	16 (40.0)
High school diploma or GED	25 (14.4) [−14.4]	114 (4.0)	14 (56.0)	24 (16.6) [−12.2]	14 (58.3)	10 (41.7)
Some college or college graduate	44 (14.70 [−6.0]	28 (63.6)	16 (36.4)	39 (15.0) [−4.2]	28 (71.8)	11 (28.2)
**Employment^e^**						
Unemployed	59 (14.0) −9.7]	32 (54.2)	27 (45.8)	51 (16.4) [−10.2]	29 (56.9)	22 (43.1)
Employed part-time	25 (13.2) [−6.8]	15 (60.0)	10 (40.0)	25 (−7.5) [16.2]	16 (64.0)	9 (36.0)
Employed full-time	23 (−7.5) (15.8)	17 (73.9)	6 (26.1)	26 (10.2) [−7.1]	19 (73.1)	7 (26.9)
**Self-rated health**						
Excellent or very good	55 (14.0) [−8.0]	32 (58.2)	23 (41.8)	54 (14.5) [−8.2]	34 (62.7)	20 (37.0
Good	36 (14.5) [−6.6]	25 (69.4)	11 (30.6)	30 (16.2) [−7.7]	20 (66.7)	10 (33.3)
Fair or poor	6 (8.10) [−12.3]	3 (50.0)	3 (50.0)	8 (11.2) [−11.7]	5 (62.5)	3 (37.5)
**Self-efficacy composite score, n (mean score) [SD],^f^**	NA	66 (41.3) [8.2]	45 (44.9) [7.4]	NA	66 (40.6) [8.0]	38 (43.9) [7.4]
**Level of engagement^g^**						
<9 Lessons at 16 wk	37 (−3.9) [11.7]	30 (81.1)	7 (18.9)	35 (‑5.0) [10.5]	29 (82.9)	6 (17.1)
≥9 Lessons at 16 wk	74 (‑10.7) [14.6]	36 (48.7)	38 (51.4)	69 (‑10.3) [16.6]	37 (53.6)	32 (46.4)
**Baseline body mass index **						
Overweight (25–<30)	35 (‑8.2) [9.5]	19 (54.3)	16 (45.7)	26 (−7.8) [12.5]	14 (53.9)	12 (46.2)
Obese (≥30)	73 (‑8.6) [16.1]	44 (60.3)	29 (39.7)	73 (‑8.7) [16.0]	49 (67.1)	24 (32.9)

Abbreviations: GED, general education diploma; NA, not applicable; SD, standard deviation; T1, data collected at approximately 6 months after baseline or 24–35 weeks; T2, data collected at approximately 12 months after baseline or 47–65 weeks.

a Values are number of participants (mean lb) [SD lb] unless otherwise noted.

b Values are number (percentage) of participants unless otherwise noted.

c For T1 and T2, percentages in ≤5% and >5% columns may not add to 100% because of rounding and missing data.

d Education: mean weight loss at T1, *P* = .05, determined by ANOVA.

e Employed part-time, work <40 hours/week; Employed full-time, work ≥40 hours/week.

f Self-efficacy composite score for diet and physical activity: T1, *P* = .02 and T2, *P* = .04, determined by *t* test.

g Engagement: T1, mean weight loss, *P* =.02, determined by χ^2^; percent body weight loss T1, *P*=.001 and T2, *P*=.003, determined by χ^2^ test.

Analysis of HbA_1c_ was limited to study participants who had HbA_1c_ measurements at baseline and T1 (n = 97) or baseline and T2 (n = 91). Participants remained in the prediabetes range from baseline to T1 (5.8% to 5.7%; *P* =.14) and T2 (5.7% to 6.0%; *P* < .001). Among participants who lost more than 5% of baseline weight at T1, their HbA_1c_ level decreased from an average of 5.8% at baseline to 5.6% at T1(*P* = .009), but no significant difference was observed at T2. For those who lost 5% or less of baseline weight, HbA_1c_ levels were the same at T1 as baseline but increased at T2 (from 5.8% at baseline to 6.1% at T2; *P* = .006).

### Comparison group analysis

We found no overall difference in HbA_1c_ between the intervention (n = 95) and control (n = 95) group (0.07%, *P* =.22). The comparison group analysis was limited to the T1 follow-up and included 109 participants (Figure). Although the comparison group also required 3 measurements of BMI and HbA_1c_ within 12 months, we were able only to obtain data close to T1 follow-up and not for T2. Because we identified each control participant on an exact match for sex, we found no differences in sex between the intervention group and control group (81% female in both groups). Mean age was 49.5 years for the intervention group and 50.6 years for the control group. At baseline, BMI was 34.8 for the intervention group and 33.3 for the control. At T1, among control group participants, we found no significant changes in BMI (33.3 at baseline to ‒32.5 at T2) or in HbA_1c_. The mean time between baseline and follow-up for the control group was 33 weeks for BMI and 37 weeks for HbA_1c_, compared with 29 weeks on average for both measurements for the intervention group. A *t* test comparison showed a 3.3% (SD, 7.2%; *P* = .001) difference in the mean percentage change in BMI between the intervention and control group at T1.

### Multivariate regression analysis 

Some sociodemographic variables, such as race/ethnicity and education, were associated with significant weight change from baseline to T2 (models 1 and 2). Compared with non-Hispanic white participants, Hispanic/Latino participants were more likely to gain weight (7.2%, 95% CI, 2.5%–11.8%) and had lower odds of having more than 5% weight loss (odds ratio [OR], 0.14; 95% CI, 0.03−0.72). Less than college education was significantly associated with weight loss (−5.7%; 95% CI, −9.1 to −2.3) and higher odds of having more than 5% weight loss (OR, 4.1; 95% CI, 1.1–15.6). Also, participants completing 9 or more lessons had higher odds of having more than 5% weight loss (OR, 5.0; 95% CI, 1.3–19.1). For model 3 showing predictors of engagement, Hispanic/Latino participants (OR, 0.11; 95% CI, 0.02–0.65) and participants who reported better health at baseline were less likely to complete 9 or more lessons (OR, 0.52; 95% CI, 0.27–0.99) (Table 3).

**Table 3:** Multivariate Regression Analysis of Participant Weight Change and Engagement

Characteristic	Model 1^a^^,^ b % Weight Change at T2 OLS: Coefficient Estimates (95% CI)	Model 2^a^^,^ b >5% Weight Change at T2 Logit: Odds Ratio (95% CI)	Model 3^a^^,^ b Engagement at T2 Logit: Odds Ratio (95% CI)
**Age** c	0.12 (−0.05 to −0.3)	0.94 (0.9 to −1.00)	1 (0.9 to −1.1)
**Sex**			
Male	1 [Reference]	1 [Reference]	1 [Reference]
Female	0.86 (−2.8 to 4.5)	1.02 (0.3 to 4.1)	1.49 (0.4 to 5.2)
**Race/ethnicity**			
Non-Hispanic white	1 [Reference]	1 [Reference]	1 [Reference]
Hispanic/Latino	7.17 (2.5 to −11.8)	0.14 (0.03 to −0.7)	0.11 (0.02 to −0.65)
Other	1.97 (−2.7 to −6.6)	0.42(0.09 to −2.1)	0.35 (0.06 to −2.1)
**Insurance status**			
Medicaid or Medi-Cal	1 [Reference]	1[Reference]	1[Reference]
Other programs for uninsured	−0.05 ( −3.5 to 3.4)	1.38 (0.4 to 5.1)	2.89 (0.9 to 9.3)
**Education**			
Some high school, GED, or high school graduate	−5.72 (−9.1 to −2.3)	4.14 (1.1 to −15.6)	0.57 (0.2 to 1.9)
Some college or college graduate	1 [Reference]	1 [Reference]	1 [Reference]
**Employment**			
Unemployed	−3.24 (‑7.1 to –0.6)	4.17^d^ (1.0 to 17.1)	0.76 (0.2 to 2.9)
Employed part-time	‑0.55 (−4.8 to 3.7)	1.72 (0.4 to 8.2)	0.96 (0.2 to 4.3)
Employed full-time	1 [Reference]	1 [Reference]	1 [Reference]
**Level of engagement**			
<9 Lessons	1 [Reference]	1 [Reference]	NA
≥9 Lessons	‑3.00 (‑6.3 to –0.3)	5.01 (1.3 to 19.1)	NA
**Self-rated health**	‑1.35 (‑3.3 to 0.6)	1.33 (0.7–2.7)	0.52 (0.27 to .99)
**Self-efficacy** composite score^d^	‑0.15 (‑0.4 to –0.1)	1.04 (1.0 to 1.1)	1.07 (1.0 to 1.2)

a Abbreviations: CI, confidence interval; GED, general education diploma; NA, not applicable; OLS, ordinary least squares.

b Model 1, OLS using percentage change in weight as a continuous variable; Model 2, logit model of weight loss, defined as ≤5% or >5% weight loss from baseline; Model 3, logit model of engagement, defined as <9 lessons or ≥9 lessons.

c Age is a continuous variable in years.

d Self-efficacy composite score for diet and physical activity: T1, *P* = .02 and T2, *P* = .04, determined by *t* test.

## Discussion

Our findings reveal that an online format for diabetes prevention and lifestyle modification can benefit many low-income patients who are at risk of diabetes and served by safety-net programs. Among our group of low-income participants who met the criteria for prediabetes, most continued the trial 1 year beyond enrollment. More than one-half were highly engaged, completing at least 9 weekly lessons of the 16-week intensive program.

Recent data suggest that the vast majority of the US population has access to various types of mobile technology, regardless of income. Many low-income individuals use the technology of mobile phones, and other devices have become less costly and more widespread (28). Less than 10% of our enrollees failed to complete the first required step of creating their program profile, indicating that comfort or access to digital technology was not a major barrier for people who want to improve their health.

The intervention helped people reduce their body weight from baseline by about 4%. Approximately 41% of the intervention group lost more than 5% of their baseline weight at T1 and 37% at T2. Results revealed that a digital prevention program can lead to successful weight loss. These results in weight loss are comparable to previous study findings that examined DPP participation of non-Medicaid beneficiaries at mixed income levels (9,29). The sustainability of weight loss for up to about 12 months in the intervention group (in contrast to the control group, in which we observed no change) indicates the effectiveness of the intervention in engaging populations at risk of diabetes to help them achieve their weight loss goals and reduce disease risk.

Our study has several limitations. We chose the study design of a nonrandomized, matched controlled trial to pragmatically assess the effectiveness of the DPP because there were no alternative programs readily available for the targeted population with prediabetes. A more rigorous selection of the control group, rather than the historical comparison group, would improve our ability to rule out regression to the mean and other factors that drive change and to help us clearly understand temporal changes in health outcomes. Additionally, because we did not conduct intent-to-treat analysis, our findings reflect data for participants who completed either follow-up at T1, T2, or both. Our conclusions are generalizable only to people who are likely to remain engaged. Nonetheless, the relative value of additional efficacy data on the DPP from highly structured randomized trials is debatable, relative to the value of more real-world tests of the program in underserved populations.

Another limitation is that we do not know why participants dropped out of the study or were lost to follow-up. When an intervention such as the DPP is offered to Medicaid beneficiaries and other safety-net populations, the expectation is that there will be a fair amount of attrition, given the high churn rate known among Medicaid enrollees as a result of competing life events, such as dealing with employment and family issues.

Although the in-person DPP is available to low-income populations, barriers to participation exist, such as time constraints, transportation, childcare, or other logistical factors that impede attendance in location-based meetings. Therefore, consideration must be given to other platforms, including digitally delivered DPPs that can remove some barriers and improve access and engagement. As providers and health policy makers consider options to prevent diabetes, this study shows that a digitally delivered intervention can be an effective option for this population, and people should be given choices of and access to digital diabetes prevention solutions to improve their health.
